# A Case of Suspected Metastatic Lung Cancer With Delayed Neurologic Presentation

**DOI:** 10.7759/cureus.73913

**Published:** 2024-11-18

**Authors:** Abiola Kehinde, Rithvik Rai, Pankaj Pankaj

**Affiliations:** 1 Endocrinology and Diabetes, Hinchingbrooke Hospital, Huntingdon, GBR; 2 Internal Medicine, Hinchingbrooke Hospital, Huntingdon, GBR

**Keywords:** brain metastasis, cavernoma, metastatic lung cancer, neurological deficit, seizures, unexplained weight loss

## Abstract

This case report details the clinical journey of an 81-year-old male presenting with significant weight loss, seizures, dysphagia, and rapidly progressive right-sided hearing loss. Initially, he experienced a tonic-clonic seizure, prompting an extensive diagnostic workup. Imaging studies, including CT and MRI, revealed multifocal cortical lesions, initially misinterpreted as cavernous hemangiomas. Subsequent evaluations confirmed the presence of a malignant mass in the right lung with extensive pulmonary metastasis. The diagnosis of metastatic lung cancer was established, leading to a multidisciplinary approach focused on palliative care due to the patient's poor prognosis and the complexity of his symptoms. This case underscores the importance of recognizing neurological manifestations in patients with underlying malignancies and the need for integrated care in such complex scenarios.

## Introduction

The intersection of neurological symptoms and systemic malignancies poses a significant challenge in clinical practice. Patients with underlying cancer can present with a variety of neurological manifestations, including seizures, altered mental status, and focal neurological deficits. This complexity necessitates a high index of suspicion and thorough diagnostic evaluation to differentiate between primary neurological disorders, paraneoplastic syndromes, and direct metastatic involvement [1].

In this case, we present an 81-year-old male with a history of rapid weight loss, new-onset seizures, dysphagia, and hearing loss, all of which ultimately led to the diagnosis of metastatic lung cancer. The initial workup raised concerns for both central nervous system metastasis and potential primary lung pathology, highlighting the intricate relationship between cancer progression and neurological health. This report aims to contribute to the understanding of how neurological symptoms can signify advanced malignancy and the critical role of a multidisciplinary approach in managing such patients.

## Case presentation

An 81-year-old male was admitted to the hospital following a tonic-clonic seizure lasting approximately 30 seconds, witnessed by his wife. The patient reported no preceding aura, postictal confusion, or tongue biting, although he experienced three episodes of vomiting after the seizure. In the four weeks leading up to his admission, he reported a significant weight loss of approximately 10 pounds, accompanied by poor appetite and night sweats. Additionally, the patient noted a rapid decline in hearing, particularly in the right ear, prompting an ENT evaluation and subsequent steroid treatment, although the underlying etiology remained unclear.

**Figure 1:**
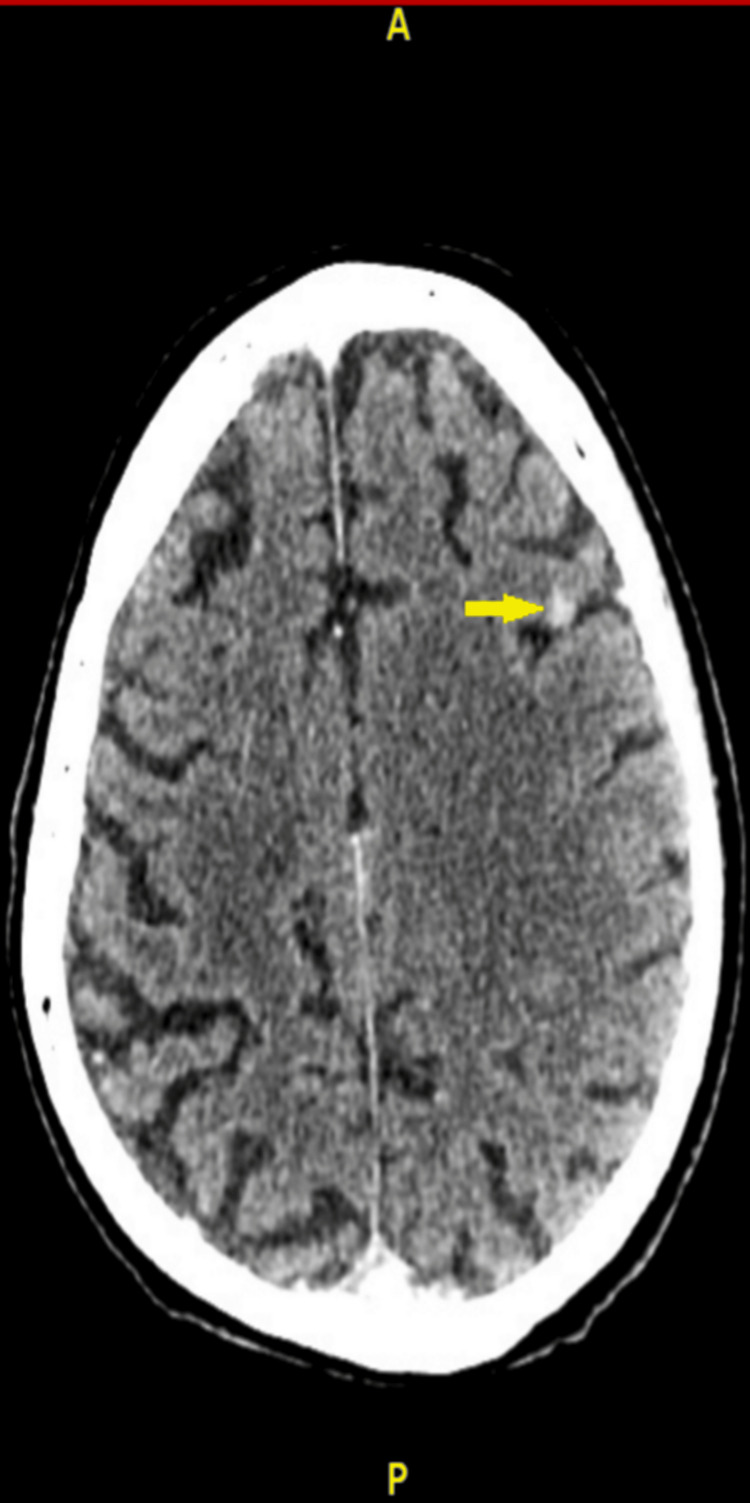
CT scan showing multifocal subcortical hyperdensity (yellow arrow).

The patient's medical history included chronic kidney disease (CKD) stage 3 and coronary artery bypass grafting, with notable occupational exposure to asbestos. Socially, he lived independently with his wife, was a former smoker (quit 40 years ago), and consumed moderate amounts of alcohol. Initial diagnostic imaging included a CT scan of the head, revealing multifocal subcortical hyperdensities (Figure 1).

**Figure 2:**
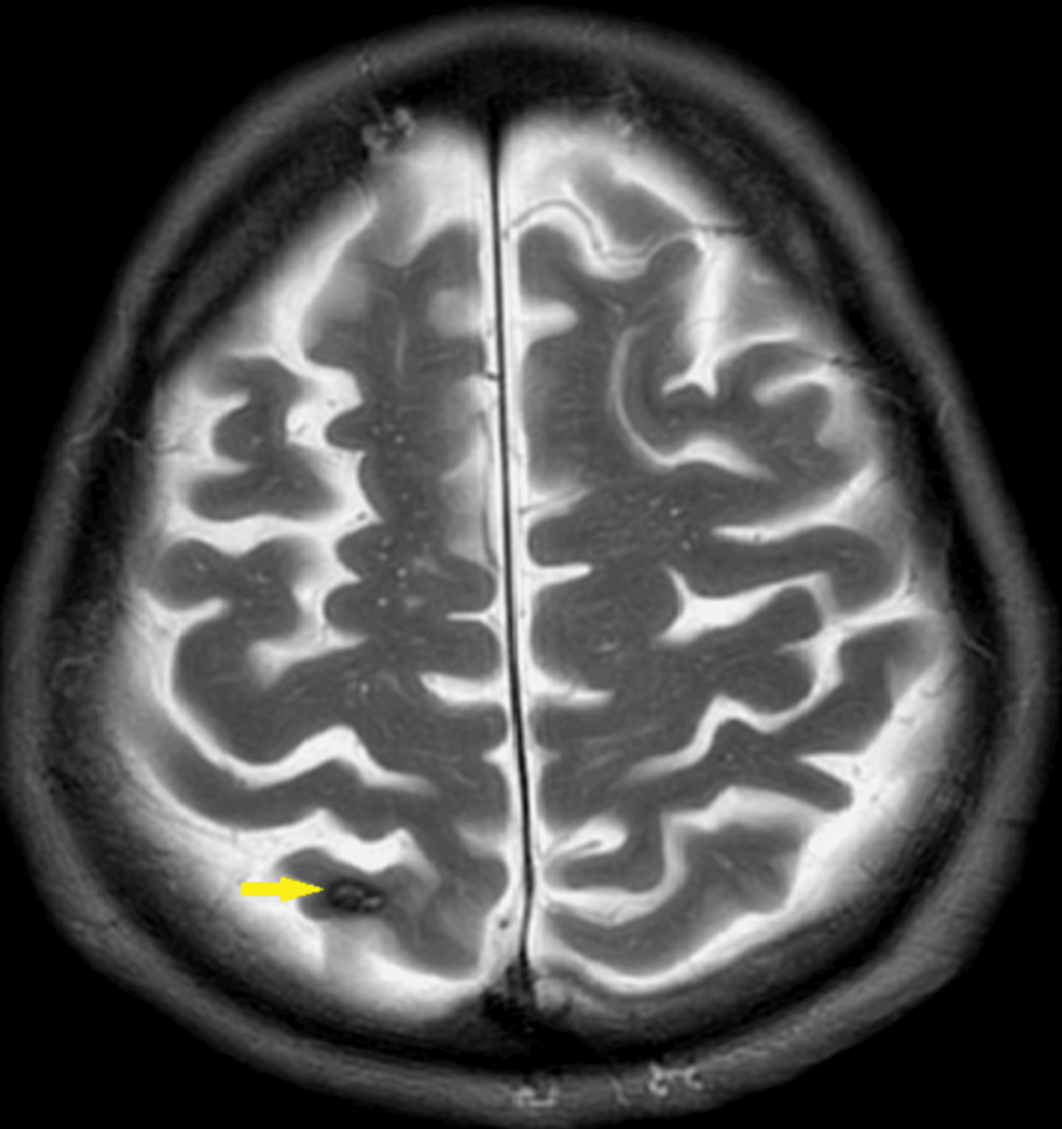
MRI head showing feature suggestive of cavernous hemangioma (yellow arrow).

The absence of acute intracranial hemorrhage and the presence of global involutional changes raised the suspicion for possible metastatic lesions. An MRI conducted the same day confirmed multiple cortical lesions with a hemorrhagic component, initially interpreted as cavernous hemangiomas (Figure 2). However, given the clinical context, metastasis could not be ruled out.

**Figure 3:**
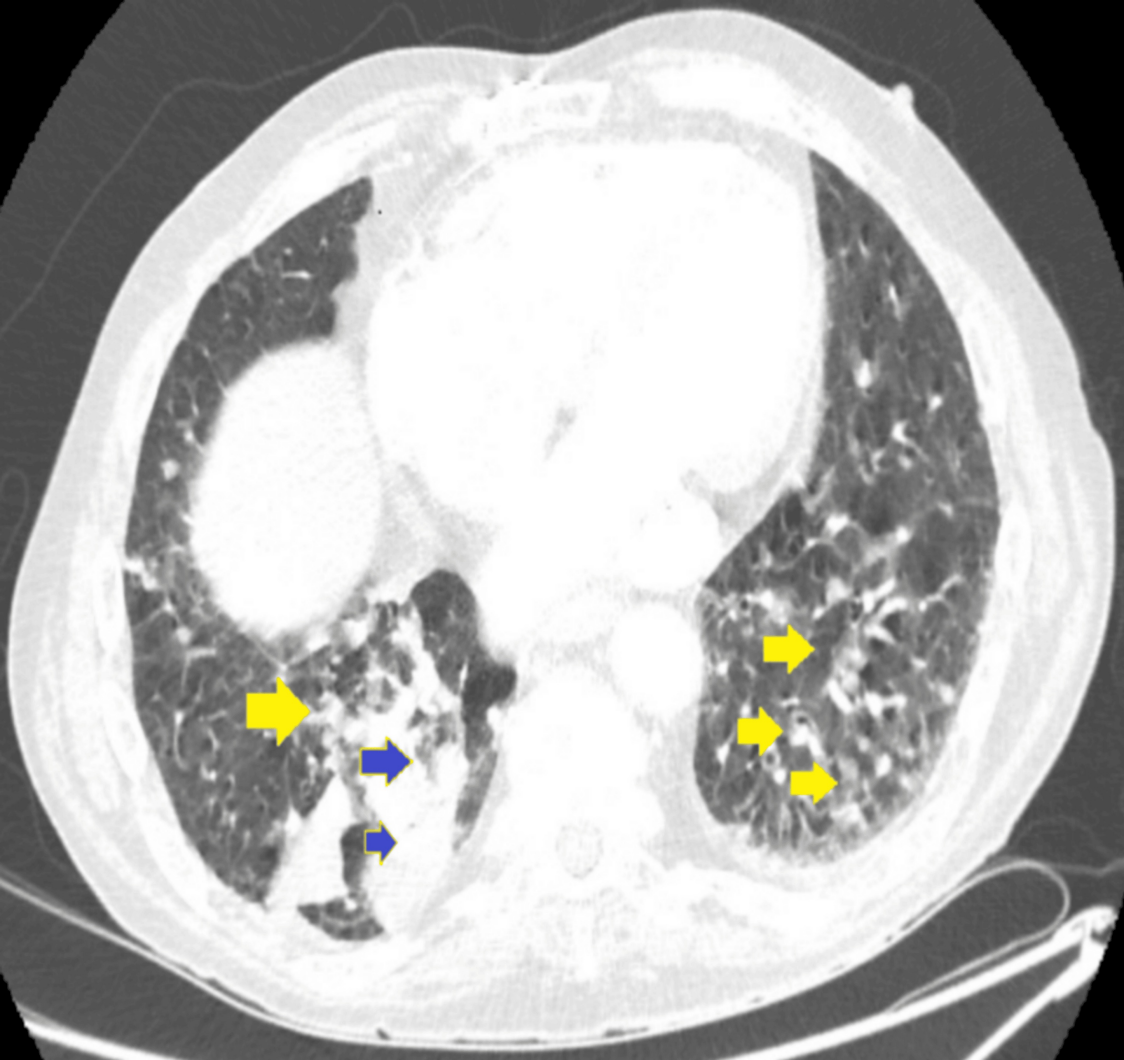
CT thorax abdo pelvis showing primary lung malignancy with extensive bilateral pulmonary metastases. 1. Yellow arrows show extensive bilateral pulmonary metastases. 2. Blue arrows show a malignant-looking mass in the right lower lobe measuring approximately 52 x 45 mm.

On day 3 of hospitalization, a CT scan of the chest and abdomen demonstrated a malignant-appearing mass in the right lower lobe, measuring approximately 52 x 45 mm, alongside extensive lung metastasis (Figure 3). The findings suggested a primary lung malignancy with widespread metastatic disease, leading to a working diagnosis of metastatic lung cancer.

Neurological evaluation

Throughout his hospitalization, the patient developed new neurological symptoms, including diplopia and a right foot drop, prompting further evaluation. An MRI of the spine revealed extensive degenerative changes but no significant bony metastatic disease. The patient's seizures were managed with Levetiracetam, and he reported no further seizure activity.

Gastrointestinal concerns

Due to significant dysphagia, the patient was made nil by mouth (NBM) and transitioned to nasojejunal feeding. An OGD performed revealed mild gastritis, with further evaluations indicating concerns for aspiration pneumonia.

## Discussion

This case emphasizes the critical need for an integrated approach in diagnosing and managing patients with concurrent neurological symptoms and malignancies. The development of seizures in patients with malignancies can occur through various mechanisms, including direct tumor infiltration, paraneoplastic syndromes, or metabolic disturbances related to cancer [2]. In this patient, the seizures were likely attributed to the metastatic involvement of the central nervous system, as evidenced by imaging findings.

The initial misinterpretation of the brain lesions as cavernous hemangiomas underscores the complexity of diagnosing intracranial lesions in cancer patients. While cavernous hemangiomas are typically benign vascular malformations, their appearance can mimic metastatic disease on imaging, complicating the clinical picture [3]. This highlights the importance of considering a broad differential diagnosis when evaluating new neurological symptoms in cancer patients.

Furthermore, the patient's dysphagia and hearing loss also warrant discussion, as these symptoms can significantly impact quality of life and nutritional status. The management of dysphagia in patients with advanced cancer often necessitates a multidisciplinary approach involving gastroenterology, neurology, and palliative care teams [4,5]. In this case, the decision to provide nasojejunal feeding was crucial given the patient's severe dysphagia and risk of aspiration.

The discussion surrounding palliative care is essential in cases of advanced malignancy. Studies have demonstrated that early integration of palliative care can improve patient outcomes, including quality of life, and potentially prolong survival [6-8]. As the patient had a poor performance status and extensive disease burden, the focus shifted to managing symptoms and ensuring comfort.

## Conclusions

This case report illustrates the complex relationship between neurological symptoms and metastatic disease in lung cancer. The patient’s progression from a single seizure to a diagnosis of extensive metastatic disease emphasizes the need for thorough clinical evaluation and advanced imaging techniques. Early signs can often be misattributed, which can delay critical interventions. A multidisciplinary approach is essential for managing patients with advanced cancer and neurological symptoms. Collaboration among oncology, neurology, palliative care, and gastroenterology teams is crucial to address the diverse needs of these patients. Each specialty offers valuable expertise that enhances symptom management and overall quality of life.

Palliative care plays a key role in supporting patients with serious illnesses. Early integration of palliative strategies can improve comfort and dignity, while also facilitating discussions about goals of care and advanced directives. This case highlights the importance of increased awareness among healthcare providers regarding the neurological complications of cancer, which can lead to timely diagnosis and intervention. By fostering a holistic and compassionate approach to care, we can better support patients navigating the complexities of metastatic disease.
